# First insight into the phylogeny of fine‐leaved *Festuca* in the Altai Mountain Country based on genome‐wide genotyping

**DOI:** 10.1002/ece3.9943

**Published:** 2023-04-02

**Authors:** Elizaveta A. Kriuchkova, Evgenii Baiakhmetov, Marcin Nobis, Polina D. Gudkova

**Affiliations:** ^1^ Research Laboratory ‘Herbarium’ National Research Tomsk State University Tomsk Russia; ^2^ Department of Botany, Institute of Biology and Biotechnology Altai State University Barnaul Russia; ^3^ Institute of Botany, Faculty of Biology Jagiellonian University Kraków Poland; ^4^ Center for Integrative Conservation, Xishuangbanna Tropical Botanical Garden Chinese Academy of Sciences Menglun China

**Keywords:** Altai Mountain Country, DArTseq, distribution, fescues, hybridization, phylogeny

## Abstract

*Festuca* is one of the largest genera within the Poaceae family. Molecular phylogenies demonstrate that *Festuca* s.l. comprises two groups: broad‐ and fine‐leaved species. The latter is the species‐richest and taxonomically complicated group due to being paraphyletic. Here, we provide the first insight into the phylogeny of 17 fine‐leaved species of Altai fescues. Based on genome‐wide genotyping, the examined taxa were divided into three markedly differentiated clusters. The first cluster comprises species from the *F. rubra* complex, the second cluster includes the *F. brachyphylla* complex, and the third cluster contains taxa from the groups *F. ovina*, *F. valesiaca*, and *F. kryloviana*. Importantly, we detected a complex genetic pattern within the groups of *F. valesiaca* and *F. kryloviana*. Moreover, our findings underline a discrepancy between morphological and molecular data for some species distributed within the Altai Mountain Country. We suggest that in order to validate the current findings on the fine‐leaved fescues, additional comprehensive research including morphological, karyological, and molecular methods is required. Nonetheless, our work provides a baseline for further investigations on the genus and studies on the floral diversity of Asia.

## Graphical abstract

The fine‐leaved fescues are the richest and the most complex group of grasses with unknown phylogenetic relationships. We analyzed 17 species of the genus from the territory of the Altai Mountain Country. Based on genome‐wide genotyping, the examined taxa were divided into three markedly differentiated clusters. The current revision revealed a complex genetic pattern among the clusters. Moreover, we questioned the presence of some species in the territory under study.

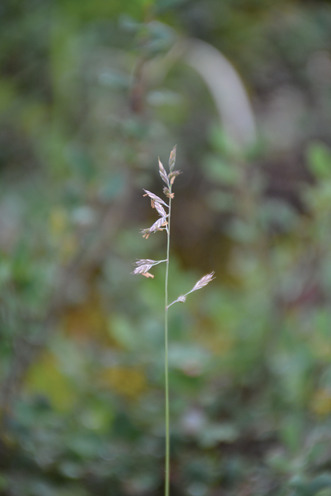

## 1 INTRODUCTION

*Festuca* L. (fescue) is one of the largest genera within the Poaceae family. It consists of approximately 450–636 species distributed mostly in the temperate and alpine zones of both hemispheres (Alexeev, 1980; Clayton & Renvoize, 1986; Lu et al., 2006; POWO, 2022; Soreng et al., 2022; Tzvelev, 1976; Watson & Dallwitz, 1992). Classification of *Festuca* has undergone significant revision in recent decades. Since the beginning of the XXI century, the widespread uptake of molecular sequencing methods has provided many new insights into the relationships within *Festuca* s.l. Recent phylogenetic analyses, based on multiple nuclear and chloroplast DNA markers, have documented the polyphyly of *Festuca* s.l. (Catalán et al., 2004, 2006, 2007; Gallaher et al., 2022; Inda et al., 2008; Torrecilla & Catalán, 2002; Torrecilla et al., 2003, 2013). Additionally, it has been estimated that approximately 70% of fescue taxa are polyploids with a wide range of ploidy levels, spanning from diploid to dodecaploid species (Bednarska & Brazauskas, 2017; Dubcovsky & Martinez, 1992; Hunziker & Stebbins, 1987; Šmarda, 2006; Šmarda et al., 2005; Šmarda & Stancik, 2006).

A range of molecular studies based on nuclear and chloroplast markers revealed the divergence of the tribe Loliinae into two well‐developed clades with broad‐leaved and fine‐leaved species (Catalán et al., 2004, 2006, 2007; Inda et al., 2008; Moreno‐Aguilar et al., 2020; Torrecilla et al., 2003, 2013; Torrecilla & Catalán, 2002). In the Altai Mountain Country (AMC, Altai, or Altai Mountains), the broad‐leaved clade comprises the genera *Drymanthele* Krecz. & Bobrov, *Schedonorus* (P. Beauv.) Peterm, and *Leucopoa* (Griseb.) Hack. The fine‐leaved group encompasses the genus *Festuca*, which is divided into sections based on the leaf blade's anatomical structure, degree of the leaf sheath closedness, pubescence degree of the ovary, adherence of the palea to the grain, and hilum characters (Alexeev, 1984; Ospina‐González et al., 2015; Pawlus, 1985; Tzvelev & Probatova, 2019).

High morphological variability is a common phenomenon observed in species representing the genus *Festuca*. However, it has also been noticed that certain characters, such as plant height, length of the lemma and awn, as well as leaf blade width, may be linked and predetermined by the ploidy level of particular fescue individuals (Alexeev, 1980; Martinez‐Sagarra et al., 2021), just as it is commonly observed in other plant species (Meeus et al., 2020; Nobis et al., 2022; Soltis et al., 2014; Stebbins, 1971). Thus, the ploidy seems to be a very important character in the taxonomy of *Festuca* (Enushchenko & Probatova, 2020; Lewitsky & Kuzmina, 1927; Litardiere, 1923; Oliveira et al., 2008; Wilkinson & Stace, 1991). Most fescue species of the Altai Mountains are polyploids. Currently, several ploidy levels are known in *F. rubra* L. (2*n* = 6*x* = 42, 8*x* = 56), *F. kryloviana* Reverd. (2*n* = 4*x* = 28, 6*x* = 42), *F. pseudovina* Hack. ex Wiesb. (2*n* = 2*x* = 14, 6*x* = 42), *F. lenensis* Drobow (2*n* = 2*x* = 14, 6*x* = 42), and *F. valesiaca* Gaud. (2*n* = 2*x* = 14, 6*x* = 42; Chepinoga et al., 2010; Probatova & Sokolovskaya, 1980; Šmarda, 2008; Šmarda et al., 2005), while *F. brevissima*, *F. musbelica*, and *F. ovina* are only diploids (2*n* = 2*x* = 14; Alexeev et al., 1990; Probatova, 1979; Probatova et al., 2013; Probatova & Sokolovskaya, 1980; Tzvelev & Probatova, 2019). *Festuca ovina* s.l. is considered to have four or more ploidy levels (2*n*, 4*n*, 6*n*, 8*n* or less 3*n*, 5*n* or 7*n*; Qiu et al., 2021). In Central Asia, the species was divided into two subspecies that later were raised to the rank of species: diploid *F. ovina* and tetraploid *F. sphagnicola* B. Keller (Enushchenko & Probatova, 2020). Unfortunately, there are still a lot of species of fescues with unknown ploidy levels, for instance, *Festuca borissii* Reverd., *F. kurtschumica* Alexeev, *F. kuprijanovii* Chusovljanov, and *F. saurica* Alexeev.

Interspecific hybridization is another quite frequent phenomenon that is observed in fine‐leaved fescues and plays an important role in species formation within the genus *Festuca* (Ardenghi et al., 2011; Bednarska, 2009; Bednarska & Brazauskas, 2017; Gutiérrez Villarías et al., 1992). The taxonomical difficulties in *Festuca* are the result of the morphological similarity of species, and the identification of hybrids based on morphological and anatomical characters is very hard, especially in complexes of closely related and morphologically similar taxa. Thus, molecular and karyological data are currently the only reliable means for identifying species and detecting gene flow (Chusovlyanov & Kotukhov, 2006; Šmarda & Kočí, 2003; Tzvelev & Probatova, 2019).

In the area of Altai Mts, the fine‐leaved fescues comprise two sections: *Aulaxyper* Dumort. (*F. rubra* group) with two species and *Festuca* (*F. ovina* group) with 17 species (Chusovlyanov & Kotukhov, 2006). Morphologically, they are characterized by the perennial caespitose and rare rhizomatous or stoloniferous and (2–)3–7(−11)‐floret spikelets, a dorsally rounded lemma with 3–5 veins, and a linear hilum (Ospina‐González et al., 2015; Tzvelev, 1976; Tzvelev & Probatova, 2019). Nevertheless, there are still no molecular studies on the Altai fescues that can support the diversity and delimitation of these species. Thus far, phylogenetic research is based on nuclear ITS (ITS1‐ 5.8S‐ITS2) and the plastid *trn*TL (*trn*T‐*trn*L intergenic spacer) and *trn*LF (*trn*A‐Leu, *trn*L‐*trn*F intergenic spacer, *trn*A‐Phe) loci (Catalán et al., 2004, 2006, 2007; Inda et al., 2008; Torrecilla et al., 2003, 2013; Torrecilla & Catalán, 2002). Currently, new high‐throughput techniques such as RADseq, GBS, and DArTseq have provided a solid tool to solve complex taxonomic problems related to hybridization and genetic variation in many wild plant genera (Baiakhmetov et al., 2021; Davey & Blaxter, 2010; Xu et al., 2017).

In this study, we aim to (1) assess genetic structure within fine‐leaved fescues of the AMC using a genome‐wide genotyping technique, (2) evaluate whether hybridization occurs between the taxa, and (3) discuss morphological and anatomical characters of these species.

## 2 METHOD

### 2.1 Plant material

We analyzed 17 species of 19 *Festuca* from the AMC. Two taxa, *F. lenensis* and *F. pseudosulcata*, are not presented in the study due to the lack of well‐preserved herbarium material. A total of 72 samples were either collected in the field or obtained from herbarium materials preserved at ALTB, KRA, KUZ, LE, and TK. Herbaria acronyms used follow Thiers (2021, continuously updated http://sweetgum.nybg.org/ih/). A complete list of taxa and voucher information can be found in Appendix A. All samples were studied by the authors. The specimens were identified according to the multiple keys (Alexeev, 1990; Darbyshire & Pavlick, 2007; Lu et al., 2006; Skvortzov, 1964; Tzvelev, 1976; Tzvelev & Probatova, 2019). Additionally, all samples were compared with the type specimens stored at LE, TK, KEW, GB, K, BM, LINN, W, and LAU. The examined species represented the section *Aulaxyper* (two taxa) and the section *Festuca* (15 taxa). We used one individual of *F. albifolia* and *F. pseudovina*; two of *F. kuprijanovii*, *F. musbelica*, and *F. richardsonii*; three of *F. borissii* and *F. saurica*; four of *F. sphagnicola, F. tschujensis*, and *F. brevissima*; and six of *F. rupicola*. For polymorphic species, we selected more samples, namely *F. rubra* (seven specimens), *F. kryloviana* (10 specimens), and *F. valesiaca* (14 specimens). The majority of specimens were sampled from the Russian part of the AMC, while *F. musbelica* and *F. tschujensis* (three specimens) were selected from Mongolia. *Festuca saurica* is an endemic of Kazakhstan and was selected from the Saur ridge. Additionally, samples of polymorphic and widespread species were selected from Poland (*F. rupicola* and *F. valesiaca*) and Kazakhstan (*F. valesiaca*). The taxon names were adopted from the WCSP (2019) and IPNI (2021).

### 2.2 DNA extraction, amplification, and DArT sequencing

Isolation of genomic DNA followed by genome complexity reduction using restriction enzymes and high‐throughput polymorphism detection (Kilian et al., 2012) were performed by Diversity Arrays Technology Pty Ltd. The resulting single nucleotide polymorphisms (SNPs) were processed in the R‐package dartR v.1.9.4 (Gruber et al., 2021) with the following parameters: (1) a scoring reproducibility of 100%; (2) SNP loci with read depth <5 or >50 were removed; (3) at least 95% loci called (the respective DNA fragment had been identified in greater than 95% of all individuals); (4) monomorphic loci were removed; and (5) SNPs that shared secondaries (had more than one sequence tag represented in the dataset) were randomly filtered out to keep only one random sequence tag. Subsequently, three approaches were used to analyze the genetic structure: (1) Unweighted Pair Group Method with Arithmetic Mean (UPGMA); (2) fastSTRUCTURE and STRUCTURE analyses; and (3) Principal Coordinates Analysis (PCoA). UPGMA cluster analyses based on the Hamming Distance with 1000 bootstrap replicates were performed in the R‐package poppr v.2.9.1 (Kamvar et al., 2014, 2015). The final UPGMA trees were visualized via iTOL v.6.3.2 (Letunic & Bork, 2021). Next, the genetic structure was investigated using fastSTRUCTURE v.1.0 (Raj et al., 2014) for all individuals. A number of clusters (K‐values) ranging from 1 to 20 were tested using the default parameters with 100 replicate runs per dataset. The most likely K‐value estimated with the best choice function and the assessment of the average proportion of membership calculated across all runs with cluster Markov Packager Across K (Clumpak; Kopelman et al., 2015) were performed via StructureSelector (Li & Liu, 2018). The output matrix for the best K‐value was plotted using the R package pophelperShiny v.2.1.0 (Francis, 2017). Additionally, to assess genetic structure at the level of particular clusters inferred with the UPGMA and fastSTRUCTURE, we used STRUCTURE v.2.3.4 (Pritchard et al., 2000) via StrAuto v.1.0 (Chhatre & Emerson, 2017). Ten replicate runs were performed for each number of clusters (K) from one to five/10 with a burn‐in of 10,000 iterations followed by 100,000 MCMC iterations. The optimal K‐value was identified based on Evanno's method of delta K statistics (Evanno et al., 2005) as implemented in Structure Harvester (Earl & von Holdt, 2012). The calculation of average proportions of membership across all runs was performed with Clumpak via StructureSelector, while the R package pophelperShiny was used to visualize the output matrices. We applied the threshold of 0.10 < q < 0.90 as the most widely utilized measure for the assessment of hybridization (Winkler et al., 2011) with q‐values >0.9 being pure species and 0.45 < q < 0.55 being F1 hybrids, while first‐ and second‐generation backcrosses with one parent were considered at q‐values 0.25 and 0.125, respectively (Beugin et al., 2018). Then, PCoAs on a Euclidean distance matrix were performed using the R‐package dartR and visualized with ggplot2 v.3.3.0 (Wickham, 2016) to show the first two components and plotly v.4.9.2 (Sievert et al., 2021) to illustrate the first three components.

## 3 RESULTS

**FIGURE 1:**
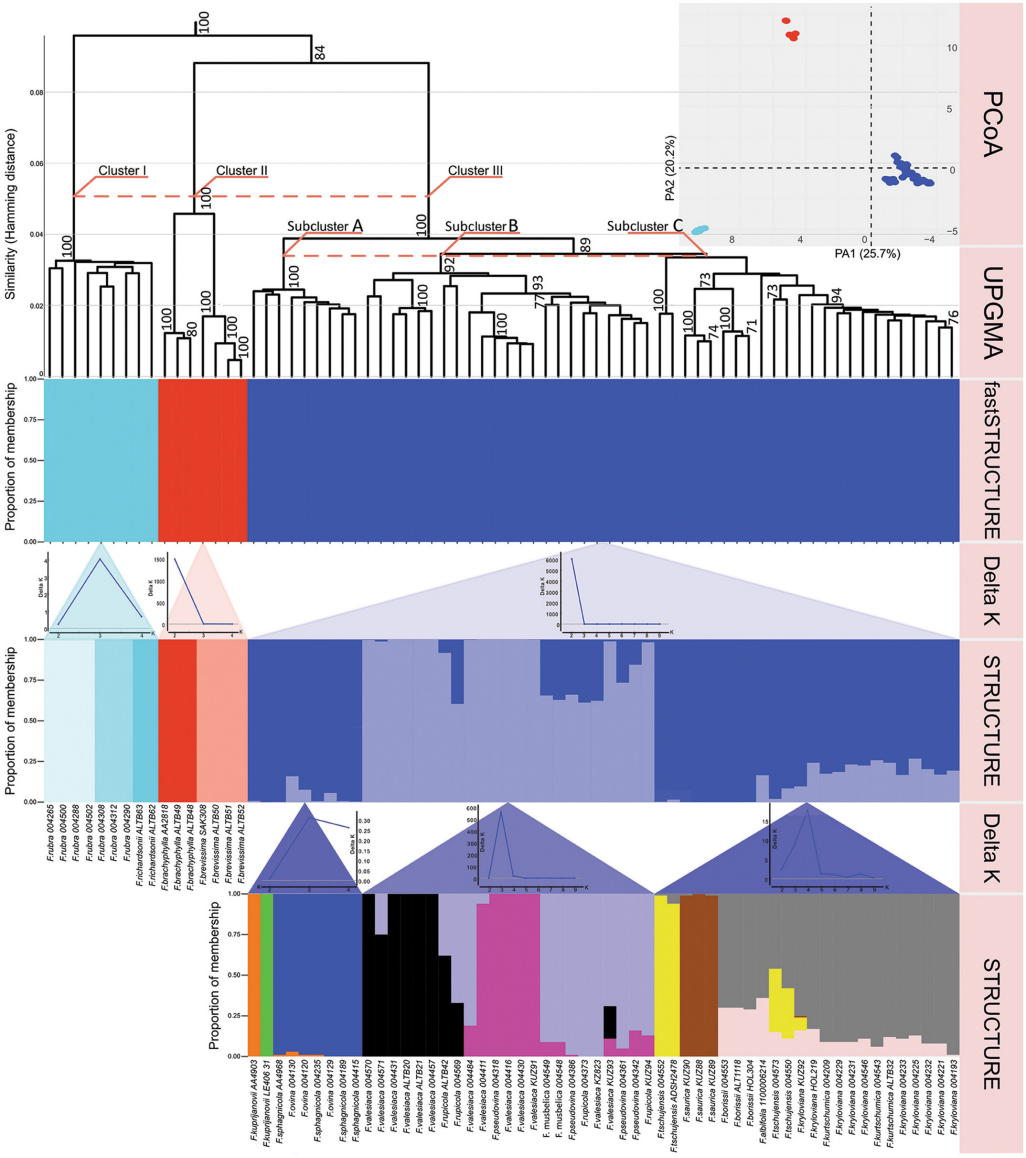
UPGMA tree reconstructed based on 1997 SNPs. Only bootstrap values > 70% obtained from 1000 replicates are shown. The *y*‐axes show the Hamming Distance, proportions of membership inferred with fastSTRUCTURE and STRUCTURE, and values of delta K obtained by Structure Harvester. The *x*‐axis for delta K represents numbers of Clusters used in STRUCTURE. Percentages in the PCoA labels represent the explained variation across the axes.

**FIGURE 2:**
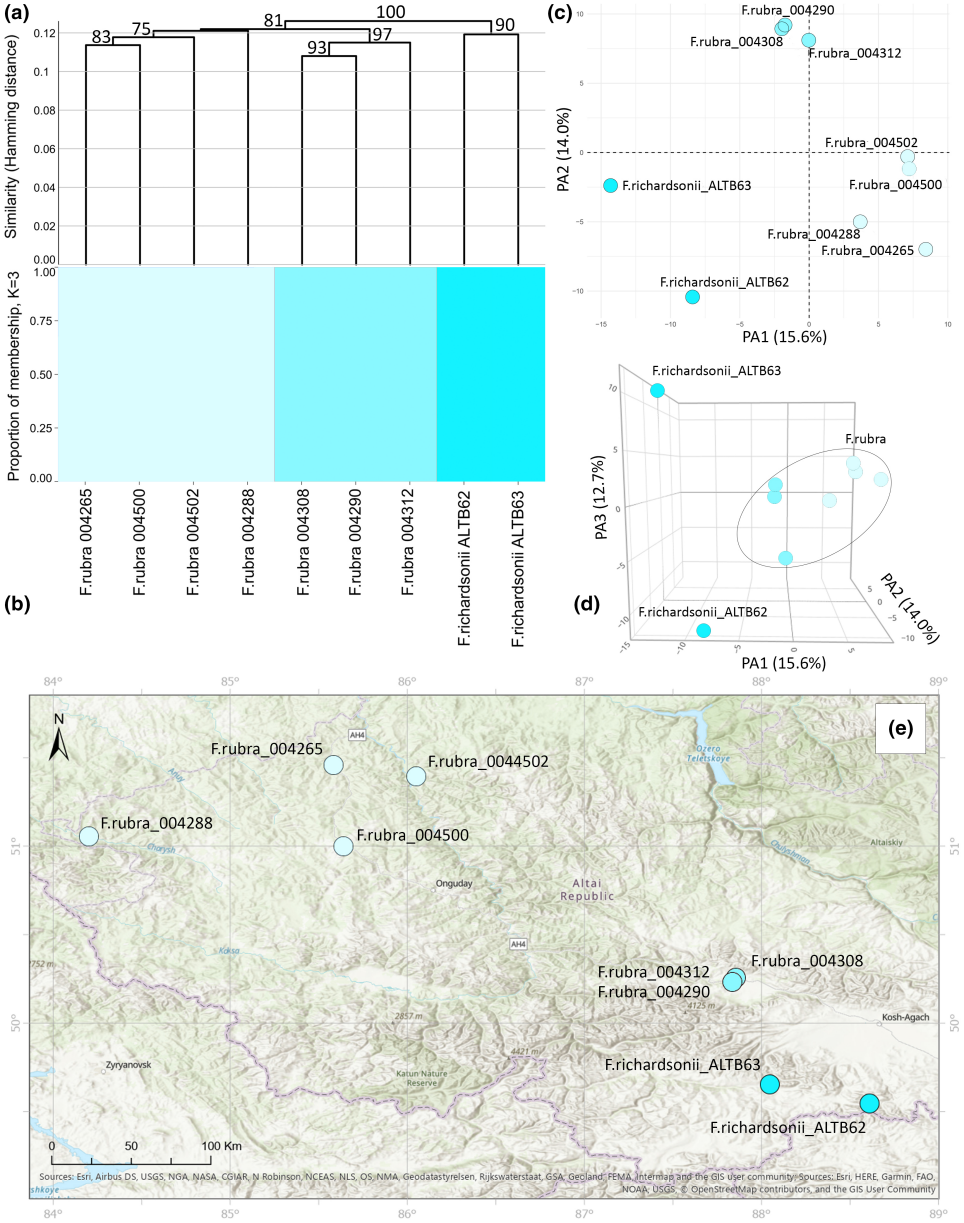
Molecular analyses of the Cluster I based on 4460 SNPs. (a) UPGMA tree. Only bootstrap values > 70% obtained from 1000 replicates are shown. The *y*‐axis shows the Hamming Distance. (b) Proportions of membership inferred with STRUCTURE for the best supported model K = 3. (c) PCoA, the first two principal coordinate axes. Percentages in the labels represent the explained variation across the axes. (d) PCoA, the first three principal coordinate axes. (e) The distribution map of the analyzed specimens.

**FIGURE 3:**
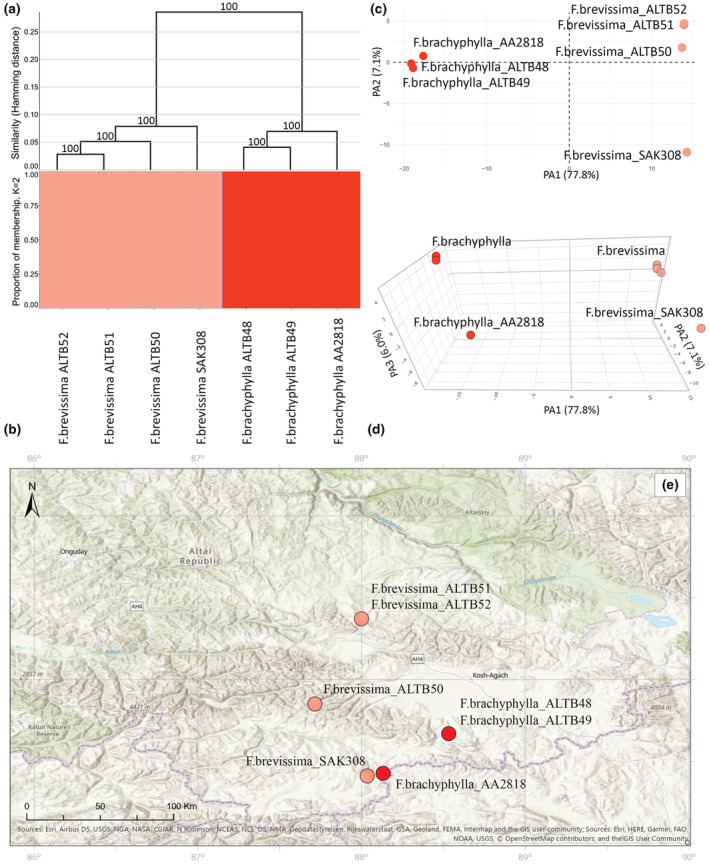
Molecular analyses of the Cluster II based on 2355 SNPs. (a) UPGMA tree. Only bootstrap values > 70% obtained from 1000 replicates are shown. The *y*‐axis shows the Hamming Distance. (b) Proportions of membership inferred with STRUCTURE for the best supported model K = 2. (c) PCoA, the first two principal coordinate axes. Percentages in the labels represent the explained variation across the axes. (d) PCoA, the first three principal coordinate axes. (e) The distribution map of the analyzed specimens.

**FIGURE 4:**
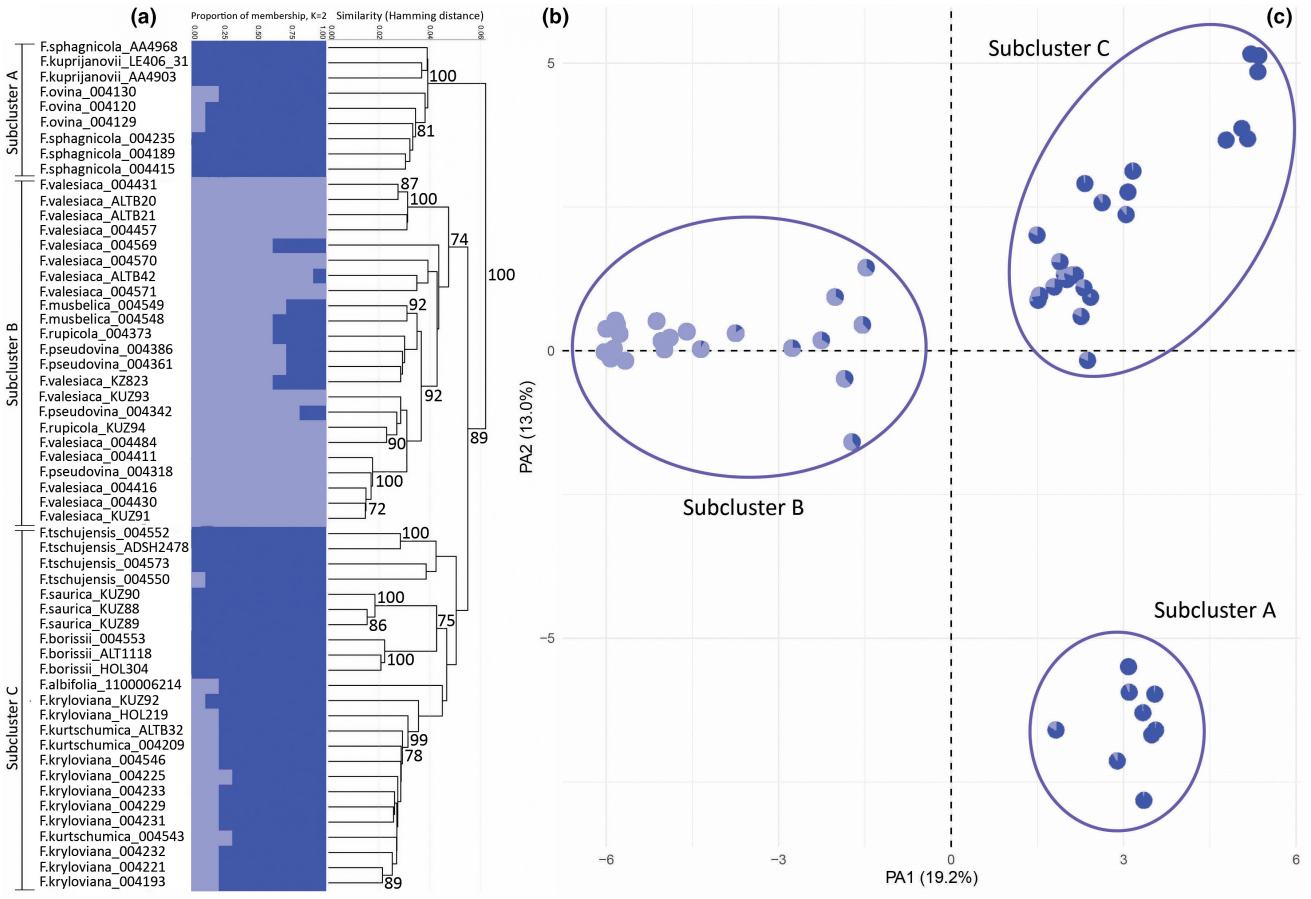
Molecular analyses of the Cluster III based on 2116 SNPs. (a) Proportions of membership inferred with STRUCTURE for the best supported model K = 2. (b) UPGMA tree. Only bootstrap values > 70% obtained from 1000 replicates are shown. The *y*‐axis shows the Hamming Distance. (c) PCoA, the first two principal coordinate axes. Percentages in the labels represent the explained variation across the axes. The pie charts represent the proportions of membership established by STRUCTURE for the best K = 2.

**FIGURE 5:**
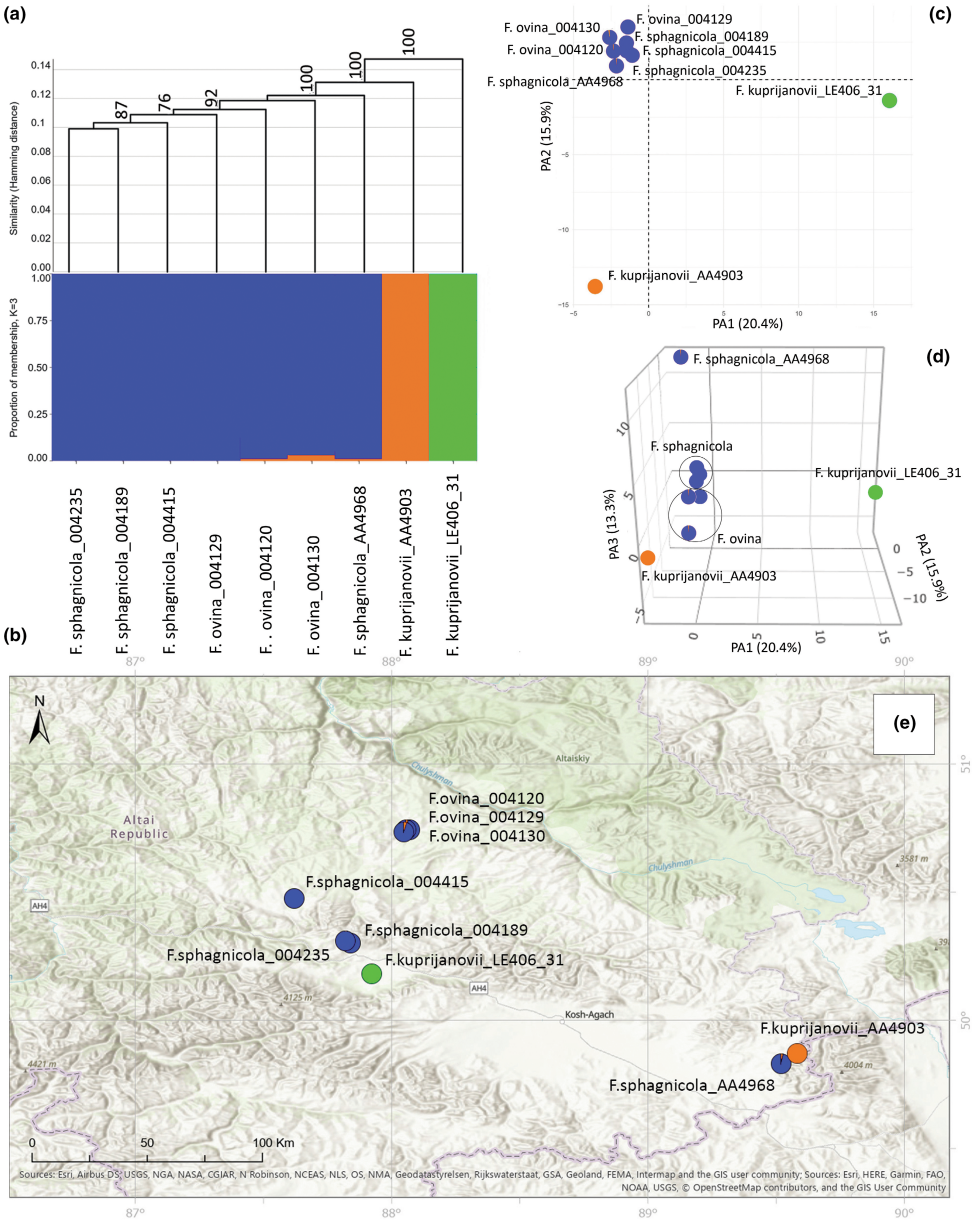
Molecular analyses of the Subcluster A based on 2118 SNPs. (a) UPGMA tree. Only bootstrap values > 70% obtained from 1000 replicates are shown. The *y*‐axis shows the Hamming Distance. (b) Proportions of membership inferred with STRUCTURE for the best supported model K = 3. (c) PCoA, the first two principal coordinate axes. Percentages in the labels represent the explained variation across the axes. The pie charts represent the proportions of membership established by STRUCTURE for the best K = 3. (d) PCoA, the first three principal coordinate axes. (e) The distribution map of the analyzed specimens.

**FIGURE 6:**
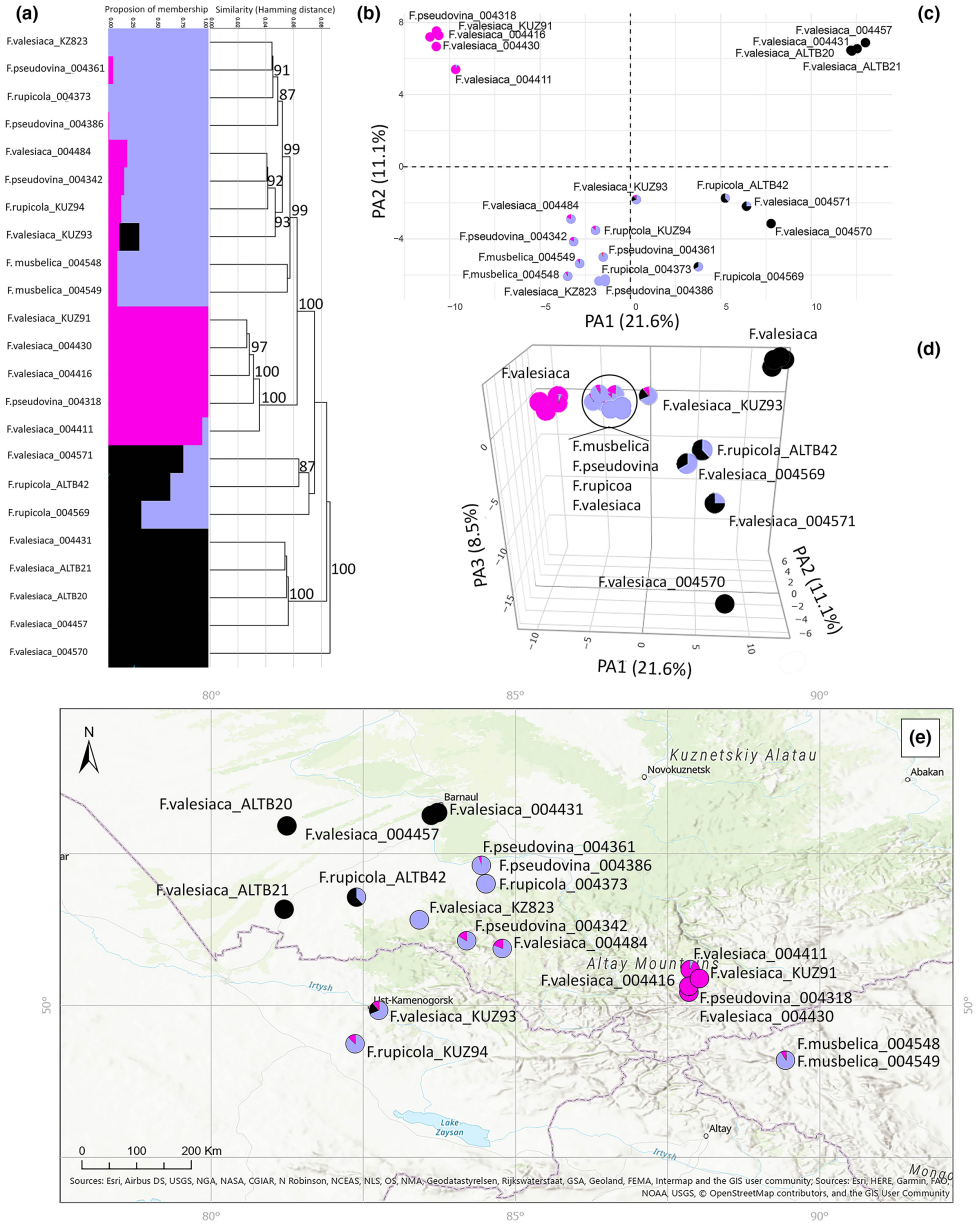
Molecular analyses of the Subclaster B based on 5417 SNPs. (a) Proportions of membership inferred with STRUCTURE for the best supported model K = 3. (b) UPGMA tree. Only bootstrap values > 70% obtained from 1000 replicates are shown. The *y*‐axis shows the Hamming Distance. (c) PCoA, the first two principal coordinate axes. Percentages in the labels represent the explained variation across the axes. The pie charts represent the proportions of membership established by STRUCTURE for the best K = 3. (d) PCoA, the first three principal coordinate axes. (e) The distribution map of the analyzed specimens.

**FIGURE 7:**
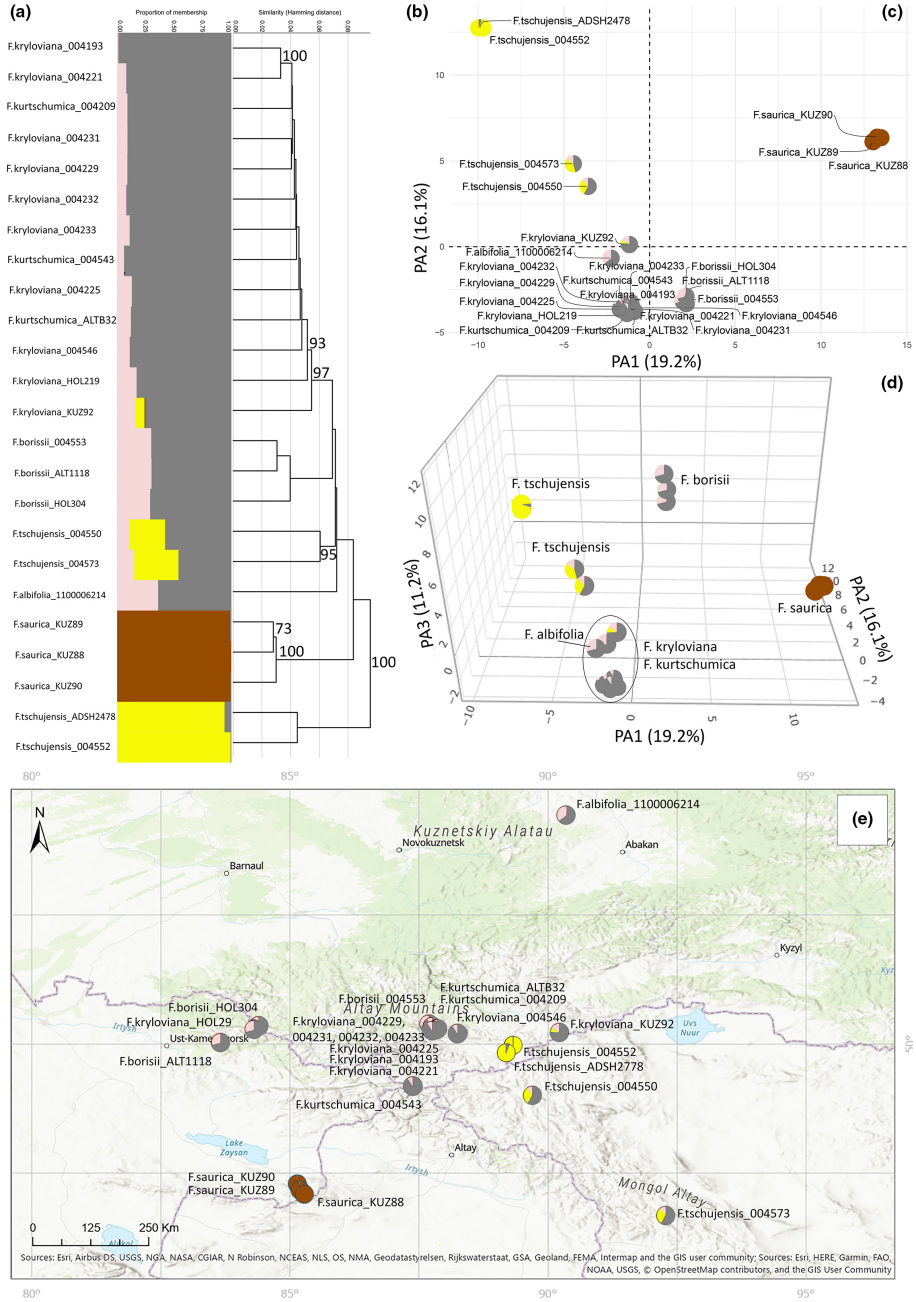
Molecular analyses of theSubcluster C based on 3516 SNPs. (a) Proportions of membership inferred with STRUCTURE for the best‐supported model K = 4. (b) UPGMA tree. Only bootstrap values > 70% obtained from 1000 replicates are shown. The *y*‐axis shows the Hamming Distance. (c) PCoA, the first two principal coordinate axes. Percentages in the labels represent the explained variation across the axes. The pie charts represent the proportions of membership established by STRUCTURE for the best K = 4. (d) PCoA, the first three principal coordinate axes. (e) The distribution map of the analyzed specimens.

A total of 1997 SNPs were retained after the filtering steps for all analyzed individuals. All three analyses, UPGMA, PCoA, and fastSTRUCTURE, revealed three markedly differentiated Clusters, corresponding to the sectional division (Figure 1). Cluster I corresponds to the section *Aulaxyper* with 2 species (*F. richardsonii* and *F. rubra*); Clusters II and III correspond to the section *Festuca* with 15 species (*F. albifolia*, *F. borissii, F. brevissima, F. brachyphylla, F. kryloviana, F. kuprijanovii, F. kurtschumica*, *F. musbelica*, *F. ovina, F. pseudovina*, *F. rupicola*, *F. saurica*, *F. sphagnicola*, *F. tschujensis*, and *F. valesiaca*). Moreover, to verify whether each inferred Cluster can be differentiated further, we performed additional analyses using UPGMA, STRUCTURE, and PCoA (Figures 2, 3, 4, 5, 6, 7).

Cluster I was analyzed separately with 4460 SNPs. All three analyses revealed the separation into three genetic groups (Figure 2a–c) that corresponded to the geographical populations (Figure 2e). Importantly, the low value of delta K = 4 (Figure 1) indicates that posterior probability differences among the different Ks are low. Thus, these specimens have weak genetic differentiation. In addition, a clear dispersal of the groups could be seen in the three‐dimensional plot, where differences between the studied species are explained by the third principal axis that accounts for 12.7% of the overall variation (Figure 2d, an interactive three‐dimensional plot available at https://plot.ly/~eugenebayahmetov/48/).

Subsequently, Cluster II was inferred with 2355 SNPs. First, the analyses of genetic clustering with UPGMA and STRUCTURE revealed two genetic Clusters that corresponded to two taxa: *F. brachyphylla* and *F. brevissima* (Figure 3a,b). This was supported by a high value of delta K = 1500 (Figure 1). According to the PCoA, the first three axes explained 77.8%, 7.1%, and 6.0% of the total genetic variance, respectively (Figure 3d). Despite the close location of the populations, the geographical location did not affect the results of the analyses (Figure 3e). All individuals were divided into two groups that are concordant with the results of UPGMA and STRUCTURE. Nonetheless, the sample *F. brevissima* SAK308 was comparatively distant to the remaining individuals of *F. brevissima* across the second axis, which explains only 7.1% of the overall variation (Figure 3c).

The analyses of Cluster III with UPGMA and PCoA based on 2116 SNPs revealed three Subclusters: A, B, and C (Figure 4), whereas the result of STRUCTURE detected only two pure genetic groups as well as specimens with admixed nature (Figure 4a). The first two axes of the PCoA explained 19.2% and 13.0% of the total genetic variation within the studied taxa, respectively (Figure 4c). According to the UPGMA and PCoA, Subcluster A (*F. ovina* group) includes specimens of *F. ovina*, *F. sphagnicola*, and *F. kuprijanovii*. Subcluster B (*F. valesiaca* group) includes specimens of *F. musbelica*, *F. pseudovina*, *F. rupicola*, and *F. valesiaca*. Subcluster C (*F*. *kryloviana* group) includes specimens of *F. albifolia*, *F. borissii*, *F. kurtschumica*, *F. kryloviana*, *F. saurica*, and *F. tschujensis*.

Subcluster A of Cluster III was inferred with 2118 SNPs. It includes *F. ovina*, *F. sphagnicola*, and *F. kuprijanovii*. The PCoA and STRUCTURE analyses divided this Subcluster into three genetic groups (Figure 5b,c). The low value of delta K = 0.3 (Figure 1) designates that posterior probability differences among the different Ks are low. Thereby, these specimens have weak genetic differentiation. Additionally, the UPGMA did not support such a division into three groups (Figure 5a). The first three axes of the PCoA explained 20.4%, 15.9%, and 13.3% of the total genetic variation within the studied taxa, respectively (Figure 5d). *Festuca kuprijanovii* specimens were divided into different groups that correspond to the location of the populations (Figure 5e). The first genetic group, represented by *F. kuprijanovii* (LE406_31), is distant to the remaining specimens by the first axis. The second genetic group, represented by *F. kuprijanovii* (AA4903), is distant to the first group by the first two axes and to the third group by the second axis (Figure 5c). The third group includes samples of *F. ovina* and *F. sphagnicola*. However, the specimen *F. sphagnicola* AA4968 is distant to other samples of *F. sphagnicola* by the third axis (Figure 5d, an interactive three‐dimensional plot available at https://plot.ly/~eugenebayahmetov/50/).

Subcluster B was analyzed with 5417 SNPs. UPGMA divided specimens into four major Clades (Figure 6b), whereas the STRUCTURE analysis revealed three genetic groups (Figure 6a). The STRUCTURE result corresponds to the PCoA. The first three axes explained 21.6%, 11.1%, and 8.5% of the total genetic variation within the studied taxa (Figure 6d). According to the PCoA, individuals were grouped into three markedly differentiated groups corresponding to two pure genotypes of *F. valesiaca* and a third pure genetic group representing *F. rupicola* (Figure 6c). Most genetic groups correspond to altitudinal zonation (Figure 6e). The remaining specimens were admixed between pure groups with an exception of individual KUZ93 that shared all three genetic groups. Additionally, one pure specimen of *F. valesiaca* (004570) from Poland was distant to the samples from the AMC (004457, 004431, ALTB20, and ALTB21) by the second and the third axes (Figure 6d, an interactive three‐dimensional plot available at https://plot.ly/~eugenebayahmetov/52/).

Subcluster C that was analyzed with 3516 SNPs includes six alpine and mountain‐steppe species (Figure 7). There was no correlation between the results of molecular analyses and the geographical location of the populations (Figure 7e). The STRUCTURE analysis revealed the most likely number of K value of 4, while the UPGMA disclosed three major clades (Figure 7b). The results of the UPGMA were also supported by PCoA (Figure 7c). The first three axes of the PCoA explained 19.2%, 16.1%, and 11.2% of the total genetic divergence within the studied group. Following the PCoA, specimens were grouped into three markedly differentiated groups corresponding to pure genotypes of *F. tschujensis* (004552, ADSH2478), *F. saurica*, and *F. kryloviana* (004193; Figure 7d). The remaining samples were mainly admixed between two groups: *F. kryloviana* and another “ghost” genetic cluster (Figure 7c, pink color), which was not represented by pure individuals. Moreover, two individuals of *F. tschujensis* (004573, 004550) shared three genetic clusters corresponding to pure genotypes of *F. tschujensis*, *F. kryloviana*, and the “ghost” pink cluster, while one specimen (KUZ92) shared all four genetic clusters. Moreover, the third axis of the PCoA shows that *F. borissii* is distant from the remaining admixed samples (Figure 7d, an interactive three‐dimensional plot available at https://plot.ly/~eugenebayahmetov/54/).

## 4 DISCUSSION

Оur phylogenetic analyses of 17 out of 19 fine‐leaved fescues using genome‐wide SNP genotyping reveals new insight into the phylogeny of these species. Previously, only five of them (*F. ovina*, *F. valesiaca*, *F. rubra*, *F. brevissima*, and *F. brachyphylla*) had been analyzed using a few loci, and the remaining 14 had not been investigated molecularly. This lack of data prevented a clear understanding of the relationships between these species within the genus. Genome‐wide SNP genotyping is an informative and accurate method used to analyze relationships within taxonomically complicated taxa. It is successfully applied to the study of natural hybridization in the wild (Baiakhmetov et al., 2020, 2021; Moreno‐Aguilar et al., 2020), including the cases of polyploid species, such as cotton (Hulse‐Kemp et al., 2015), oat (Tinker et al., 2009), wheat (Wang et al., 2015), and strawberry (Bassil et al., 2015). Our molecular data provided the information that allowed us to review the value of morphological characters in AMC fescue species. Furthermore, we have a detailed discussion about the correspondence between molecular and morphological data.

### 4.1 Clade composition and morphological traits

Our findings clearly demonstrate that the studied individuals can be grouped into three clusters. Cluster I includes two species, *F. rubra* and *F. richardsonii*, both belonging to the section *Aulaxyper*. *Festuca richardsonii* was a questionable species that was provided from the AMC and occurs at an altitude of 3000 m (Alexeev, 1990; Chusovljanov, 2007). Although the analyses revealed the specimens ALTB62 and ALTB63 formed separate well‐supported genetic groups, we presume that this is an indication of genetic variability at the population level, as supported by the low delta k value. Morphologically, the ALTB62 and ALTB63 specimens are distinguished from *F. rubra* by the lemma length, the awn length, and the lemma pubescence (Alexeev, 1990; Tzvelev, 1976; Tzvelev & Probatova, 2019). Furthermore, specimens of *F. richardsonii* from the Arctic and the AMC differ by the culm length 50–60 versus 10–30(−40) cm; the shape of the panicle open versus contracted; the number of spikelets on the lower panicle branches 2 or more versus 1–2 spikelets, and the awn length 1–2.3 versus 0–1(−1.5) mm, respectively. The specimens of *F. richardsonii* from the AMC belong to *F. rubra*; however, due to the latter species being extremely polymorphic, further comprehensive taxonomical revision is required.

Cluster II includes the samples of *F. brachyphylla* and *F. brevissima*, both from the section *Festuca*. These two species differ from the other Altai fescues by having shorter anthers, 0.5–1.5 versus 1.5–3 mm, respectively. The UPGMA, STRUCTURE, and PCoA confirm a strong genetic differentiation between these taxa. Morphologically, these species are easily distinguishable by the number of spikelets in the panicle <8 versus >11 spikelets, the number of spikelets on the lower branches 1–2 versus 2 and more spikelets, the panicle length 0.7–26 versus 23–55 mm, the lemma length 2.5–4 versus 4.5–5.5 mm, and the plant length up to 12 versus 10–55 cm. It is worth noticing that *F. brevissima* is a new record to the Altai Mountains and currently it is the southernmost locality within the species range. Also, *F. brevissima* is morphologically similar to another arctic species, *F. edlundiae*. However, they differ by, for example, the length and shape of сulms, the shape of glumes, and the number of chromosomes 2*n* = 2*x* = 14 versus 2*n* = 4*x* = 28 (Darbyshire & Pavlick, 2007).

Within Cluster III, Subcluster A includes specimens representing *F. kuprijanovii*, *F. ovina*, and *F. sphagnicola* characterized by a well‐defined midrib and a continuous sclerenchyma layer. *Festuca kuprijanovii* is an endemic species known only from the Altai Republic (Chusovljanov, 1998). It is closely related to *F. sphagnicola* and *F. ovina*. Following Chusovljanov (1998), *F. kuprijanovii* differs from *F. ovina* and *F. sphagnicola* by an abaxial sclerenchyma continuous ring with a thickening opposite midrib and by spikelet's color, brown versus green. The samples of *F. kuprijanovii* are divided into two genetic groups that correspond to their geographical distribution. *Festuca ovina* and *F. sphagnicola* form a common genetic group according to the PCoA and STRUCTURE. Nevertheless, *F. ovina* differs from *F. sphagnicola* by having green versus brown spikelets and a diploid versus tetraploid set of chromosomes (Alexeev, 1990; Šmarda & Kočí, 2003). Thus, due to molecular markers being used for the first time to delimitate the latter two species, we should perform additional morphological and karyological studies to verify the genetic nature of these species.

Subcluster B includes *F. pseudovina*, *F. rupicola*, *F. valesiaca*, and *F. musbelica*. The STRUCTURE analysis revealed three genetic groups that did not correspond to the taxonomic classification. The PCoA revealed two well‐defined genetic groups within *F. valesiaca* that differ in geographical distributions. The first one occurs in mountains (the Kurai ridge), while the second one grows on lowlands (the Priobskoe plateau). The third group is represented by *F. rupicola* specimens that differ from *F. valesiaca* by having leaf blades 0.5–0.8 versus (0.35)0.4–0.6 mm wide, lemma 3.8–4.5 versus (2.3)2.8–3.8 mm long, spikelets 5.5–7 versus (4)4.5–5.5(6) mm long, and 5–7 versus 5 vascular bundles. According to our analyses, individual 004342 identified by the keys (Alexeev, 1990; Lu et al., 2006; Tzvelev, 1976; Tzvelev & Probatova, 2019) as *F. pseudovina*, appeared to be of a hybrid nature between *F. rupicola* and *F. valesiaca*. Moreover, two individuals (004548 and 004549), identified as *F. musbelica*, also appeared to share genetic clusters represented by *F. rupicola* and *F. valesiaca*, although morphologically they have brown spikelets and leaf sheaths closed up to ⅓–⅟₄ of its length that are most specific for *F. musbelica* (Alexeev, 1990; Lu et al., 2006; Tzvelev, 1976; Tzvelev & Probatova, 2019). Additionally, six more specimens, preliminarily determined either as *F. rupicola* or *F. valesiaca*, also had a complex genetic pattern. Thus, our results support the prior research regarding hybridization in *Festuca*. We treat the current findings with caution due to the sample size being limited and we cannot deny the presence of incomplete lineage sorting and introgression that also may influence the complex genetic structure of these specimens. Nonetheless, previously, hybrids have been already detected between *F. varia* subsp. *eskia* (*F. eskia* Ramond) and *F. varia* subsp. *pumila*; *F. eskia* and *F. gautieri* (Hackel) K. Richter. (*F. x souliei*) in Europe (Gutiérrez Villarías et al., 1992); *F. polesica* and *F. arundinaceae*, *Festuca* ×*polovina* in Ukraine (Bednarska, 2009); *Festuca rubra* and *Vulpia myuros* in Italy (Ardenghi et al., 2011); *F. galiciensis* × *F. rupicola* in Ukraine (Bednarska & Brazauskas, 2017). Thus, the complex genetic structure in this group brings difficulties with the proper taxonomic identification of specimens. Hence, it is possible that some of the described taxa within fine‐leaved fescues with weak morphological differences may in fact represent nothospecies. Lastly, the issues in this group can also be associated with different ploidy levels among different species and among populations of the same species. For instance, *F. valesiaca* is diploid and hexaploid (Arndt, 2008; Probatova et al., 2008; Šmarda, 2008; Šmarda et al., 2005), while *F. rupicola* and *F. pseudovina* are hexaploids (Alexeev et al., 1988; Šmarda et al., 2005). Whereas, samples of a hybrid origin may have the same or different ploidy levels in comparison with parental species (Bednarska & Brazauskas, 2017).

Subcluster C consists of *F. albifolia*, *F. borissii*, *F. kryloviana*, *F. kurtschumica*, *F. tschujensis*, and *F. saurica* species that are characterized by leaf sheaths closed for ¼–⅘ of its length and three sclerenchyma strands. However, our results detected only three genetically separated species, namely *F. tschujensis*, *F. kryloviana*, and *F. saurica*. The latter taxon is an endemic species from the Saur ridge of Kazakhstan (Alexeev, 1976; Chusovljanov, 2007; Chusovlyanov & Kotukhov, 2006). Additionally, we detected a few specimens with a hybrid nature, for example, samples 004573 and 004550; however, morphologically, they correspond to the description of *F. tschujensis*. *Festuca albifolia* is also morphologically similar to *F. tschujensis* (Alexeev, 1990; Tzvelev, 1976; Tzvelev & Probatova, 2019). Nonetheless, our study demonstrates that a sample (1100006214) determined as *F. albifolia* appeared to be genetically closer to the *F. kryloviana* group that also includes specimens of *F. borissii* and *F. kurtschumica*. *Festuca kryloviana* exhibits high variability of morphological and anatomical characters that may depend on a different number of chromosomes (2*n* = 4*x* = 28 or 6*x* = 42; Chepinoga et al., 2010; Probatova & Sokolovskaya, 1980). Moreover, the result of the PCoA revealed that *F. borissii* is distinguished from *F. kryloviana* by the third axis. The ranges of these species overlap in the territory from the mountains of southern Kazakhstan to the northern part of the AMC. Morphologically, these species can be well‐separated by the plant life form, the shape of the leaf blade cross‐section, the lemma length, the awn length, and the length of the closed portions of the sheaths (Chusovlyanov & Kotukhov, 2006; Tzvelev & Probatova, 2019). In addition to the known species, STRUCTURE revealed an unknown genetic group found in *F. albifolia*, *F. borissii*, *F. kryloviana*, and *F. kurtschumica*. Possibly, this “ghost” cluster represents a species that is not included in the analysis. Alternatively, it may be inherited from an extinct species. In order to resolve the phylogenetic relationships within the Altai fescues, our further research should be supplemented with morphological and karyological studies due to different ploidy levels playing an important role in the description of the species. Importantly, we ought to enlarge the sample size of the studied taxa to verify if morphological plasticity is constant within their geographic ranges.

## 5 CONCLUSIONS

In this study, we provide the first insight into the genetic structure within fine‐leaved fescues of the AMC and a baseline for further investigations of the genus. Altai fescues were classified into five groups, two clusters and three subclusters, of closely related species. We found that Altai *F. richardsonii* is conspecific with *F. rubra. Festuca brachyphylla*, *F. brevissima*, *F. borissii*, and *F. saurica* appear to be well‐separated and genetically distinctive. Due to the usage of genome‐wide genotyping, we were able to detect a complex genetic pattern in the *F. valesiaca* and *F. kryloviana* complexes. The inconsistency between morphological characters and molecular data for some species distributed within the Altai Mountains is the basis for evaluating the usefulness of the diagnostic characters currently being used to identify these species. We reckon that a combination of morphological, karyological, and molecular analyses is needed to resolve the remaining questions related to the interspecific relationships within the genus *Festuca*.

## AUTHOR CONTRIBUTIONS

**Elizaveta Alexandrovna Kriuchkova:** Visualization (equal); writing – original draft (equal). **Marcin Nobis:** Investigation (equal); methodology (equal); writing – original draft (equal); writing – review and editing (equal). **Evgenii Baiakhmetov:** Methodology (equal); visualization (equal); writing – original draft (equal); writing – review and editing (equal). **Polina Dmitrievna Gudkova:** Investigation (equal); resources (lead); writing – original draft (equal); writing – review and editing (equal).

## FUNDING INFORMATION

This study was supported by the Tomsk State University Development Programme (Priority‐2030).

## CONFLICT OF INTEREST STATEMENT

None declared.

## Data Availability

The SNP dataset derived from the DArTseq pipeline in the genlight format is available via Figshare repository, https://doi.org/10.6084/m9.figshare.19329812.v1. The high‐quality Figures are available via Figshare repository, https://doi.org/10.6084/m9.figshare.21914157.v1.

## APPENDIX A

**List of samples used in the study.**

**Table:**

Taxa	Voucher	Country
*Festuca kryloviana*	Revushkin A.S., Kobylenko S.V., Ebel A.L. Festuca_kryloviana_004545 (ALTB)	Russia, Altai Republic
*Festuca kryloviana*	Ebel A.L. Festuca_kryloviana_004546 (ALTB)	Russia, Altai Republic
*Festuca kryloviana*	Kriuchkova E.A, Ryzhakova D.D., Gudkova P.D. Festuca_kryloviana_004233 (TK)	Russia, Altai Republic
*Festuca kryloviana*	Kriuchkova E.A, Ryzhakova D.D., Gudkova P.D. Festuca_kryloviana_004231 (ALTB)	Russia, Altai Republic
*Festuca kryloviana*	Kriuchkova E.A, Ryzhakova D.D., Gudkova P.D. Festuca_kryloviana_004232 (TK)	Russia, Altai Republic
*Festuca kryloviana*	Kriuchkova E.A, Ryzhakova D.D., Gudkova P.D. Festuca_kryloviana_004225 (TK)	Russia, Altai Republic
*Festuca kryloviana*	Kriuchkova E.A, Ryzhakova D.D., Gudkova P.D. Festuca_kryloviana_004211 (TK)	Russia, Altai Republic
*Festuca kryloviana*	Kriuchkova E.A, Ryzhakova D.D., Gudkova P.D. Festuca_kryloviana_004229 (TK)	Russia, Altai Republic
*Festuca kryloviana*	Kriuchkova E.A, Ryzhakova D.D., Gudkova P.D. Festuca_kryloviana_004193 (TK)	Russia, Altai Republic
*Festuca valesiaca*	Kriuchkova E.A, Ryzhakova D.D., Gudkova P.D. Festuca_valesiaca_004457 (TK)	Russia, Altai Territory
*Festuca valesiaca*	Kriuchkova E.A, Ryzhakova D.D., Gudkova P.D. Festuca_valesiaca_004431 (TK)	Russia, Altai Territory
*Festuca valesiaca*	Kriuchkova E.A, Ryzhakova D.D., Gudkova P.D. Festuca_valesiaca_004411 (TK)	Russia, Altai Republic
*Festuca valesiaca*	Kriuchkova E.A, Ryzhakova D.D., Gudkova P.D. Festuca_valesiaca_004416 (ALTB)	Russia, Altai Republic
*Festuca valesiaca*	Kriuchkova E.A, Ryzhakova D.D., Gudkova P.D. Festuca_valesiaca_004430 (TK)	Russia, Altai Republic
*Festuca valesiaca*	Kriuchkova E.A, Ryzhakova D.D., Gudkova P.D. Festuca_valesiaca_004484 (TK)	Russia, Altai Republic
*Festuca valesiaca*	Kopytina T., Stas E., Sosnovskaya E. Festuca_valesiaca_ALTB21 (ALTB)	Russia, Altai Territory
*Festuca valesiaca*	SHmakova A.I., Vaganov A.V., Kopytina T.M., Borovikov V. Festuca_valesiaca_ALTB20 (ALTB)	Russia, Altai Territory
*Festuca valesiaca*	SHmakov A.I, Smirnov S.V., Kosachev P.A., Kechaikin A.A. Festuca_valesiaca _KZ823 (ALTB)	Russia, Altai Republic
*Festuca valesiaca*	Nobis M. Festuca_valesiaca_004571 (KRA)	Poland
*Festuca valesiaca*	Nobis M. Festuca_valesiaca_004570 (KRA)	Poland
*Festuca ovina*	Kriuchkova E.A, Ryzhakova D.D., Gudkova P.D. Festuca_ovina_004130 (TK)	Russia, Altai Republic
*Festuca ovina*	Kriuchkova E.A, Ryzhakova D.D., Gudkova P.D. Festuca_ovina_004129 (TK)	Russia, Altai Republic
*Festuca ovina*	Festuca_ovina_004120 (ALTB)	Russia, Altai Republic
*Festuca sphagnicola*	Kriuchkova E.A, Ryzhakova D.D., Gudkova P.D. Festuca_sphagnicola_004415 (ALTB)	Russia, Altai Republic
*Festuca sphagnicola*	Kriuchkova E.A, Ryzhakova D.D., Gudkova P.D. Festuca_sphagnicola_004235 (TK)	Russia, Altai Republic
*Festuca sphagnicola*	Kriuchkova E.A, Ryzhakova D.D., Gudkova P.D. Festuca_sphagnicola_004189 (TK)	Russia, Altai Republic
*Festuca kuprijanovii*	SHmakov A.I., Smirnov S. Festuca_kuprijanovii_AA4903 (ALTB)	Russia, Altai Republic
*Festuca kuprijanovii*	Kutafev V., Eremina T. Festuca_kuprijanovii_LE406_31(LE)	Russia, Altai Republic
*Festuca kurtschumica*	SHmakov A.I. Festuca_kurtschumica_ALTB32 (ALTB)	Russia, Altai Republic
*Festuca kurtschumica*	Kamelin R.V., SHmakov A.I., Smirnov S. Festuca_kurtschumica_004543 (ALTB)	Russia, Altai Republic
*Festuca kurtschumica*	Kriuchkova E.A, Ryzhakova D.D., Gudkova P.D. Festuca_kurtschumica_004209 (TK)	Russia, Altai Republic
*Festuca musbelica*	Festuca_ musbelica _004548 (ALTB)	Mongolia
*Festuca musbelica*	Festuca_ musbelica _004549 (ALTB)	Mongolia
*Festuca rupicola*	Kriuchkova E.A, Ryzhakova D.D., Gudkova P.D. Festuca_ rupicola _004361 (TK)	Russia, Altai Territory
*Festuca pseudovina*	Kriuchkova E.A, Ryzhakova D.D., Gudkova P.D. Festuca_pseudovina_004342 (TK)	Russia, Altai Republic
*Festuca valesiaca*	Kriuchkova E.A, Ryzhakova D.D., Gudkova P.D. Festuca_valesiaca_004318 (TK)	Russia, Altai Republic
*Festuca rupicola*	Kriuchkova E.A, Ryzhakova D.D., Gudkova P.D. Festuca_rupicola_004386 (TK)	Russia, Altai Territory
*Festuca rupicola*	Nobis M. Festuca_rupicola_004569 (KRA)	Poland
*Festuca rupicola*	Kamelin R.V., Shmakov A.I. Festuca_rupicola_ALTB42 (ALTB)	Russia, Altai Territory
*Festuca rupicola*	Kriuchkova E.A, Ryzhakova D.V., Gudkova P.D. Festuca_rupicola_004373 (ALTB)	Russia, Altai Territory
*Festuca borissii*	Speranskaya N.YU., Elesova N.V., Solomonova M.YU. Festuca_borissii_004553 (ALTB)	Russia, Altai Republic
*Festuca borissii*	Kamelin R.V., Shmakov A.I. Festuca_borissii_HOL304 (ALTB)	Russia, Altai Republic
*Festuca_kryloviana*	Kamelin R.V., Shmakov A.I. Festuca_kryloviana_HOL219 (ALTB)	Russia, Altai Republic
*Festuca borissii*	Rodionov A.V., Punina E.O. Festuca_borissii_ALT1118 (ALTB)	Russia, Altai Republic
*Festuca tschujensis*	Ebel A.L. Festuca_tschujensis_004573 (ALTB)	Mongolia, Kobdo Aimag
*Festuca tschujensis*	Rodionov A.V., Punina E.O. Festuca_tschujenssis_ADSH2478 (ALTB)	Russia, Altai Republic
*Festuca tschujensis*	Festuca_tschujenssis_004552 (ALTB)	Mongolia
*Festuca tschujensis*	Festuca_tschujenssis_004550 (ALTB)	Mongolia
*Festuca brevissima*	SHmakov A.I. Festuca_brevissima_SAK308 (ALTB)	Russia, Altai Republic
*Festuca brachyphylla*	Kucev M.G., German D.A. Festuca_brachyphylla_ALTB48 (ALTB)	Russia, Altai Republic
*Festuca brachyphylla*	Kucev M.G., German D.A. Festuca_brachyphylla_ALTB49 (ALTB)	Russia, Altai Republic
*Festuca brevissima*	Naumov I.V. Festuca_brevissima_ALTB50 (ALTB)	Russia, Altai Republic
*Festuca brevissima*	Ebel A.L. Festuca_brevissima_ALTB51 (ALTB)	Russia, Altai Republic
*Festuca brevissima*	Ebel A.L. Festuca_brevissima_ALTB52 (ALTB)	Russia, Altai Republic
*Festuca brachyphylla*	SHmakov A.I. Festuca_brachyphylla_AA2818 (ALTB)	Russia, Altai Republic
*Festuca rubra*	Kriuchkova E.A, Ryzhakova D.D., Gudkova P.D. Festuca_rubra_004265 (TK)	Russia, Altai Republic
*Festuca rubra*	Kriuchkova E.A, Ryzhakova D.D., Gudkova P.D. Festuca_rubra_004288 (TK)	Russia, Altai Republic
*Festuca rubra*	Kriuchkova E.A, Ryzhakova D.D., Gudkova P.D. Festuca_rubra_004290 (TK)	Russia, Altai Republic
*Festuca rubra*	Kriuchkova E.A, Ryzhakova D.D., Gudkova P.D. Festuca_rubra_004312 (TK)	Russia, Altai Republic
*Festuca rubra*	Kriuchkova E.A, Ryzhakova D.D., Gudkova P.D. Festuca_rubra_004500 (TK)	Russia, Altai Republic
*Festuca rubra*	Kriuchkova E.A, Ryzhakova D.D., Gudkova P.D. Festuca_rubra_004502 (TK)	Russia, Altai Republic
*Festuca rubra*	Kriuchkova E.A, Ryzhakova D.D., Gudkova P.D. Festuca_rubra_004308 (TK)	Russia, Altai Republic
*Festuca_richardsonii*	Naumov I.V., Sklozubov R.G. Festuca_richardsonii_ALTB62 (ALTB)	Russia, Altai Republic
*Festuca richardsonii*	Smirnov S.V., Naumov I.V., Zubov R.A. Festuca_richardsonii_ALTB63 (ALTB)	Russia, Altai Republic
*Festuca albifolia*	Neifeld, Kurochkina A. Festuca_albifolia_1,100,006,214 (ALTB)	Russia, KHakassia
*Festuca saurica*	Kotukhov YU. Festuca_saurica_KUZ88 (KUZ)	Kazakhstan, East Kazakhstan Region
*Festuca saurica*	Kotukhov YU. Festuca_saurica_KUZ89 (KUZ)	Kazakhstan, East Kazakhstan Region
*Festuca saurica*	Kotukhov YU. Festuca_saurica_KUZ90 (KUZ)	Kazakhstan, East Kazakhstan Region
*Festuca valesiaca*	Ebel A.L. Festuca_valesiaca_KUZ91 (KUZ)	Russia, Altai Republic
*Festuca valesiaca*	Kotukhov YU. Festuca_valesiaca _KUZ93	Kazakhstan, East Kazakhstan Region
*Festuca rupicola*	Kotukhov YU. Festuca_rupicola_KUZ94 (KUZ)	Kazakhstan, East Kazakhstan Region
*Festuca sphagnicola*	Shmakov A.I. Festuca_sphagnicola_AA4968 (ALTB)	Russia, Altai Republic
